# AhR and Cancer: From Gene Profiling to Targeted Therapy

**DOI:** 10.3390/ijms22020752

**Published:** 2021-01-13

**Authors:** Anaïs Paris, Nina Tardif, Marie-Dominique Galibert, Sébastien Corre

**Affiliations:** 1 CNRS (Centre National de la Recherche Scientifique), IGDR (Institut de Génétique et Développement de Rennes), UMR6290, University Rennes, F-35000 Rennes, France; anais.paris@univ-rennes1.fr (A.P.); nina.tardif50@gmail.com (N.T.); 2 Department of Molecular Genetics and Genomics, Hospital University of Rennes (CHU Rennes), F-35000 Rennes, France

**Keywords:** AhR transcription factor, expression, cancer, targeted therapy

## Abstract

The aryl hydrocarbon receptor (AhR) is a ligand-activated transcription factor that has been shown to be an essential regulator of a broad spectrum of biological activities required for maintaining the body’s vital functions. AhR also plays a critical role in tumorigenesis. Its role in cancer is complex, encompassing both pro- and anti-tumorigenic activities. Its level of expression and activity are specific to each tumor and patient, increasing the difficulty of understanding the activating or inhibiting roles of AhR ligands. We explored the role of AhR in tumor cell lines and patients using genomic data sets and discuss the extent to which AhR can be considered as a therapeutic target.

## 1. Introduction

The aryl hydrocarbon receptor (AhR) is a ligand-activated transcription factor that has multiple critical cellular functions [1]. It belongs to the basic helix-loop-helix/Per-Arnt-Sim (bHLH/PAS) family and is widely distributed in tissues and among species [2,3]. Evolution of the receptor in the vertebrate branch resulted in its ability to bind to a wide range of structurally diverse ligands. Indeed, AhR binds to endogenous (FICZ, kynurenine, etc.) and exogenous (TCDD, BaP, etc.) low-molecular-weight planar ligands that can exhibit tissue-specific agonist or antagonist activities [4,5]. In the absence of a ligand, AhR makes up part of a cytosolic multiprotein complex, consisting of c-Src kinase, Hsp90, and the chaperones p23 and XAP2 [6,7]. Binding of a ligand to AhR induces conformational changes, leading to dissociation of the protein complex and nuclear translocation of AhR. In the nucleus, AhR dimerizes with its partner protein AhR nuclear translocator (ARNT) and binds to xenobiotic-responsive elements (XREs) in the regulatory region of target genes, inducing their transcription [8,9].

Since the early 90s, AhR has been defined as an essential environmental sensor that enables the activation or inhibition of cellular pathways in response to a broad spectrum of ligands in a cell-type- and context-specific manner [1,10]. More recently, its role in cancer development has been demonstrated, in which it can either act as a positive or negative regulator of carcinogenesis.

Here, we summarize the role of AhR in cancer mechanisms, based on previous studies and the analysis of a set of genetic and genomic databases. Then, we discuss the conditions required to consider AhR as a therapeutic target.

## 2. Results

### 2.1. AhR Mutations, Level of Expression, and Activation in Cancer

**Figure 1:**
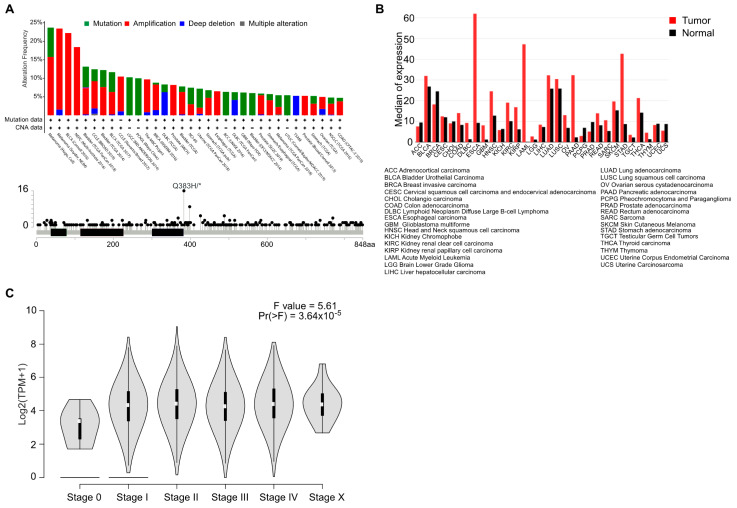
Status of the aryl hydrocarbon receptor (AhR) transcription factor status in cancer. (**A**) The frequency of alteration (top) and mutational status (bottom) of the AhR transcription factor was analyzed in all genetic and genomic data from patients or cancer cell lines from the cBioPortal for cancer genomics (http://www.cbioportal.org). AhR genetic alteration include mutation, amplification and deep deletion. (**B**) Analysis of *AhR* expression in all cancers from the TCGA compared to that in normal tissue using GEPIA 2 (Gene Expression Profiling Interactive Analysis, http://gepia2.cancer-pku.cn). (**C**) Analysis of *AhR* expression according to the staging of tumors described in 1B.

We explored the genetic landscape of *AhR* alterations in cancer by interrogating available genomic data (TCGA, Sanger, Broad, etc.) searchable on the cBioPortal for cancer genomics online platform (http://www.cbioportal.org) [11]. We recovered only a very small proportion of amplifications, mutations, or deletions of the *AhR* gene (Figure 1A, top). Only one somatic point mutation was identified with a high frequency in bladder cancer. This mutation (Q383H), located downstream of the PAS-B domain (ligand-binding domain), has not yet been functionally characterized (Figure 1A, bottom). Despite the absence of recurrent genetic abnormalities in cancer, the level of *AhR* mRNA is elevated in almost 70% of various tumor types relative to healthy tissue (Figure 1B). Indeed, *AhR* mRNA is overexpressed in breast cancer [12,13], lung cancer [14], thyroid cancer [15], and oral squamous cell carcinoma (OSCC) [16]. A high level of AhR protein has also been reported in pancreatic cancer [17], endometrial cancer [18], and meningioma [19]. Median expression of *AhR* appears elevated from stage I, independently of the tumor type, suggesting that this increased expression is an early event in many cancer (Figure 1C). Accordingly, *AhR* expression was shown to be associated with a poor prognosis in glioma [20]. On the contrary, *AhR* expression was significantly lower in primary peripheral blood chronic myeloid leukemia (CML) cells than in healthy controls supporting the notion of cell specific functions of AhR [21].

In addition to the overexpression of *AhR* mRNA and protein, the activity of the receptor has been found to be significantly elevated in various types of cancer. For example, both elevated AhR expression and activity have been observed in papillary thyroid carcinoma (PTC) [22], primary breast cancer [23], and cutaneous squamous-cell carcinoma [24]. Moreover, nuclear localization of AhR has been associated with a worse outcome for patients with high-grade anaplastic meningioma [19] or ovarian cancer [25]. In this context, Kolluri et al. widely described the role of various AhR ligands in the phenotypic control of cancer cells and tumor development [26]. Overall, it is difficult to establish a clear relationship between AhR ligands and their role in controlling proliferation, migration, and tumor cell invasion. Indeed, it appears that the consequences on tumor progression are completely different depending on the tumor type, the function of the ligand (AhR agonist or antagonist), and the cellular and protein context. Bian et al. showed that ITE (2-(1’H-indole-3’-carbonyl)-thiazole-4-carboxylic acid methyl ester), an endogenous AhR ligand, suppresses endometrial cancer cell proliferation and migration [18]. Jin et al. showed that both omeprazole and 2,3,7,8-tetrachlorodibenzo-p-dioxin (TCDD) inhibit the invasion of breast-cancer cells but only omeprazole inhibits the invasion of Panc1 pancreatic cancer cells [27]. Conversely to it, several studies have shown that AhR activation by endogenous or exogenous ligands leads to increased tumor-cell migration and aggressiveness in breast cancer [28,29] and lung-cancer cell lines exposed to kynurenine [30] and benzo(a)pyrene (BaP) [31,32]. Although the impact of AhR expression on carcinogenesis is difficult to characterize, its activation by diverse ligands and the role of various cofactors are important for determining how AhR influences tumor development and phenotype. The role of AhR ligands in controlling its activity is difficult to interpret, as activation by a single ligand (TCDD) elicits species-specific changes in gene expression. Indeed, despite relatively high conservation of AhR between species (up to 73% between humans, mice, and rats), its function is significantly different in mice, with a higher affinity for its ligand (TCDD) [33,34]. Overall, the role of AhR ligands in carcinogenesis must be approached in a tissue- and species-specific manner.

**Figure 2:**
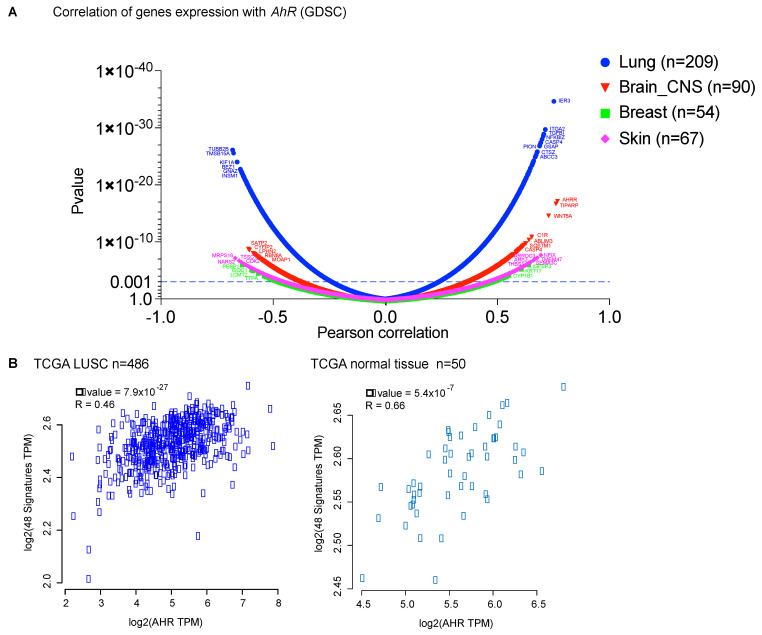
Identification of AhR correlated gene signatures in various cancers. (**A**) Volcano plots showing genes for which the expression significantly correlates with *AhR* mRNA levels (**A**) in various cancer cell lines (lung, brain-CNS (Central Nervous System), breast, skin) from the GDSC database (Genomics of Drug Sensitivity in Cancer) (https://discover.nci.nih.gov/cellminercdb). (**B**) Correlation of expression (Spearman) between *AhR* mRNA levels and genes previously identified to correlate the most positively and negatively (*n* = 48) in lung-cancer cell lines (**A**) in both datasets from the TCGA for patients in lung squamous-cell carcinoma (LUSC) tumors (*n* = 486) versus normal tissue (*n* = 50) (http://gepia2.cancer-pku.cn).

AhR is involved in the transcriptional control of many genes upon recognition of its cognate XRE-binding motifs [9,35]. This motif is highly represented throughout the genome and conserved between species [36]. Yang at al. performed genome-wide mapping and analysis of AhR-binding sites in human breast cancer cells before and after induction by TCDD using ChIP-seq analysis and identified up to 4000 AhR-bound regions [37]. In addition to AhR direct target genes, coregulated AhR genes are expected to participate in the AhR response. In this context, we analyzed the genes for which the expression correlated significantly, either positively or negatively, with *AhR* mRNA levels across tumor cell lines (lung, brain-CNS, breast, skin melanoma) of the GDSC database (Genomics of Drug Sensitivity in Cancer) using the CellMiner Cross Database web application (https://discover.nci.nih.gov/cellminercdb) [38] (Figure 2A). The expression of a large number of genes significantly correlated (*p* < 0.001) with *AhR* mRNA levels across tumor types, in particular those in the lung and brain (Figure 2A). As anticipated, they differed according to cancer type. Importantly, the *AhR* correlation signatures identified in cell lines (GDSC database) were also observed in patient tumor samples (TCGA) (Figure 2B).

### 2.2. The Paradoxical Role of AhR: Oncogene or Tumor Suppressor?

As already mentioned (Figure 1A), there is no recurrent AhR alteration in cancer. However, its involvement in carcinogenesis has been clearly established, with many studies describing its pro- or anti-tumor functions in several types of cancer [10,26,39]. This suggest that the level of AhR expression and the modulation of its activity by specific ligands may drive oncogenesis or suppress tumor development. To date, it is still not clear whether AhR ligands located in the tumor microenvironment can modulate AhR activity to the point that it influences tumor development. As the pro- and anti-tumoral roles of AhR were extensively reviewed a few years ago [10,26,39], we will focus only on the most recent data to address AhR activity in the context of such complexity.

#### 2.2.1. AhR as an Oncogene

AhR functions as a pro-tumoral factor by directly modulating the invasive properties of cancer cells. Transcriptional inhibition of *AhR* was shown to induce expression of the tumor suppressor gene *E-cadherin* (*CDH1*), reducing the mesenchymal properties of breast-cancer cell lines. In accordance, *AhR* expression was shown to correlate with an invasive transcriptomic signature, and AhR inhibition reduced the metastatic potential of breast-cancer cells in zebrafish [40]. 

Opitz et al. established that kynurenine (Kyn), a tryptophan catabolite, can bind and activate AhR [41]. Kynurenine was shown to be an endogenous oncometabolite that induces the expression of growth-controlling genes in colon- [42] and lung-cancer cells [43]. In thyroid-tumor samples, the AhR target genes *CYP1A1* and *CYP1B1* were upregulated relative to associated healthy tissue [15] and again Kyn stimulation of thyroid-cancer cell lines promoted the acquisition of an EMT program (decreased E-cadherin, and increased SLUG, *N*-cadherin, and fibronectin levels). This resulted in increased cell motility and cell invasion. Three enzymes are known to catalyze the breakdown of tryptophan into Kyn, namely tryptophan-2,3-dioxygenase (TDO), indoleamine-2,3-dioxygenase-1 (IDO1) and indoleamine-2,3-dioxygenase-2 (IDO2). IDO1 is more broadly expressed than IDO2 and has a significantly higher enzymatic activity rate, while TDO has a different distribution than IDO. In glioma, IDO1/TDO was shown to account for Kyn release and subsequent AhR-activation mediated cell motility via the expression of aquaporin 4 (AQP4) [44]. 

In addition to the Kyn-dependent pathway, AhR activation by FICZ (6-formylindolo [3,2-b]carbazole), a skin tryptophan photoproduct, was shown to promote TNFα-dependent inflammation and induce melanoma cell differentiation and the development of metastasis [45]. AhR activation by BaP has also been shown to influence the EMT through the regulation of a long non-coding RNA in non-small cell lung cancer (NSCLC) [46]. Similarly, AhR can reactivate the LINE-1 retro-transposon, silenced by DNA methylation, in breast cancer via the regulation of TGF-β signaling, promoting tumorigenesis and disease progression [47]. 

In addition to the above-mentioned role of the IDO/TDO-Kyn-AhR pathway in cancer development, many studies have demonstrated that kynurenine activation of AhR induces immunosuppressive effects, with the generation of immune-tolerant dendritic cells (DCs) and regulatory T cells. AhR is also required to induce *IDO* expression in DC. Collectively, this fosters the acquisition of a tumor microenvironment that is defective in recognizing and eradicating cancer cells [48]. 

Overall, this non-exhaustive collection of studies shows that AhR activation promotes tumor progression in various types of cancer and that the immunosuppressive properties of the kynurenine-activated AhR constitutes a highly promising axis for cancer treatment [39,49].

#### 2.2.2. AhR as a Tumor Suppressor

Despite its role as an oncogene, AhR functions as a tumor suppressor in many cancers associated with the brain and central nervous system, liver, digestive system, skin (melanoma), and reproductive tract. Such a suppressive role was uncovered using engineered mouse models in which AhR expression was abolished (*AhR* ^−/−^ mice). In this model, liver tumor formation and growth were significantly higher than in control mice, with *AhR*^−/−^ hepatocytes showing significantly higher numbers of 4N cells, increased expression of proliferative markers, and the repression of tumor suppressor genes. *AhR* silencing in this model was thus associated with cancer progression [50].

Similar results have been obtained in the context of colon cancer. Through the use of an intestinal-specific *AhR*^−/−^ mouse model, Garcia-Villatoro et al. demonstrated that expression of AhR in intestinal epithelial cells was required to reduce the formation of premalignant colon cancer lesions. Furthermore, a high-fat diet combined with loss of AhR in intestinal epithelial cells influenced the development of colorectal cancer [51]. Shiizaki et al. showed that AhR activation induces β-catenin ubiquitination and subsequent proteosomal degradation. Thus, AhR^−/−^ mice spontaneously developed cecal tumors as the result of aberrant β-catenin accumulation [52,53]. Similarly, treatment with TCDD (0.1–100 nM) diminishes colony formation and proliferation of human colorectal cancer cells [54]. 

Activation of AhR by kynurenine has also been reported to inhibit the growth of tumor cells, promote cellular differentiation, and decrease the formation of hepatic and pulmonary metastases in mice through activation of the tumor suppressor gene *KISS1* [55].

AhR has also been proposed to have a tumor suppressor function in melanoma, as its knockdown promotes primary melanoma tumorigenesis and lung metastasis in mice. In this context, AhR may antagonize the pro-tumoral effects of Aldh1a1; thus, an AhR^low^/Aldh1a1^high^ phenotype could be indicative of a poor outcome in melanoma [56,57].

Saric et al. identified AhR as a potent tumor suppressor in a SHH medulloblastoma mouse model by controlling the TGFβ/SMAD3 signaling axis to inhibit proliferation and promote the differentiation of cancer-propagating cells (CPCs) (reservoir of cells capable of tumor regeneration and relapse post-treatment) [58]. 

In glioblastoma, inhibition of AhR has been associated with activation of the CXCL12-CXCR4-MMP9 signaling pathway, involved in cell growth, invasion-migration, and cell proliferation [59]. In childhood neuroblastoma, AhR plays a protective role, as its expression correlates with a better outcome. Over-expression of AhR in pituitary adenoma (PA) cells revealed potential tumor suppressor activity independent of exogenous ligand activation by BaP [60]. 

Finally, AhR has been shown to prevent tumor development through the regulation of several tumor suppressor miRNAs (microRNAs) in breast cancer [61], prostate cancer [62], and malignant tumors of the endometrium [63]. 

Overall, these studies underscore the role of AhR as a tumor suppressor. It should be noted, however, that such a tumor suppressor function has been mostly described in mice, underscoring the specificity of AhR function between species.

### 2.3. Therapeutic Opportunities of Targeting AhR in Neoplastic Diseases

As discussed above, the role of AhR in cancer development is complex (oncogene or tumor suppressor). Nonetheless, it constitutes a promising drug target. Targeting AhR must be patient- and tumor-specific and dependent on AhR expression and activation. Three major points need to be addressed to efficiently modulate AhR activity for the treatment of neoplastic diseases. They are:

(a) To identify AhR ligands for their agonist or antagonist functions. Such ligands can be found amongst dietary molecules (flavonoids) or FDA-approved drugs. 

(b) To prevent the production (endogenous) or intake (exogenous) of oncogenic AhR activators.

(c) To prevent the interaction between oncogenic-ligands and AhR using antagonists.

Alternative AhR-targeting strategies can also be considered, such as AhR as a complementary target to increase the efficiency of cancer therapy or a means to counteract resistance mechanisms.

#### 2.3.1. AhR as a Direct Drug Target

A number of strategies have been investigated in the context of targeting AhR as a first-line treatment for cancer. Various antagonists have been tested to lower the level of *AhR* expression in the tumor when it has an oncogenic function. Conversely, other studies have aimed to promote activation of AhR through the use of agonists when the transcription factor acts as a tumor suppressor.

#### 2.3.2. Limiting Tumor Progression through AhR Activation 

AhR activity can be augmented using potent AhR agonists, but related toxicity may be an important drawback. Indeed, TCDD, the highly toxic AhR agonist, cannot be used in the clinic to specifically target AhR, despite its positive effect against breast cancer, by disrupting the CXCR4/CXCL12 pathway [64], or ovarian cancer cells [65]. Most studies have thus investigated endogenous or exogenous molecules for their ability to inhibit tumor progression.

Among the most promising molecules, ITE, an endogenous AhR agonist, reduces the aggressiveness of triple-negative breast cancer (TNBC) by downregulating JAG1-NOTCH1 signaling [66]. ITE suppresses the proliferation and migration of endometrial cancer (EC) cells in vitro and the growth of EC xenografts in mice [18]. It also suppresses the proliferation and migration of ovarian cancer cells [67]. FICZ has also been shown to have anti-proliferative and anti-migratory properties on LNCaP cells, a cell line derived from androgen-sensitive human prostate adenocarcinoma cells [68]. Finally, FICZ significantly reduces the clonogenic potential of CD34-positive cells in chronic myeloid leukemia (CML) [21].

The exogenous AhR activator, 5F 203 (2-(4-amino-3-methylphenyl)-5-fluorobenzothiazole), has shown a positive effect in several cancers. 5F 203 induces the expression of the putative tumor suppressor gene cytoglobin (*CYGB*) in TNBC [69]. It reduces in-vitro and in-vivo cell proliferation of gastric cancer [70], human renal carcinoma cells [71], and ovarian cancer cells [72]. The anti-inflammatory drug leflunomide, approved for the treatment of rheumatoid arthritis in 1998, has been shown to be an AhR agonist [73]. This molecule shows promise in cancer treatment, notably for melanoma [74,75], bladder cancer [76], and oral squamous-cell carcinoma [77]. Indirubins E804 (indirubin-3’-(2,3 dihydroxypropyl)-oximether) and 7BIO (7-Bromoindirubin-3′-oxime), synthetic derivatives of natural indirubin, activate AhR and inhibit the synthesis of important pro-inflammatory cytokines, such as IL-6 and the oncogene STAT3. They could, thus, constitute promising new treatments for glioblastoma [78].

#### 2.3.3. Limiting Tumor Progression through AhR Inhibition

When AhR has oncogenic activity or is overexpressed, the most obvious strategy is to use an antagonist. Pharmacological inhibition of AhR has been achieved using the compound 3′,4′-dimethoxyflavone (3′,4′-DMF) on breast-cancer cells, blocking formation of the nuclear AhR complex [79]. Comparatively, the specific antagonist CH-223191 reduces the clonogenic survival and invasiveness of glioma cells through control of the TGFβ pathway [80]. Since the discovery of the benefits of AhR inhibition, many studies have aimed to develop new AhR antagonists using, for example, original in vivo (zebrafish) models [81] and in silico screening [82]. Among the identified compounds, CB7993113 [82] and GNF351 [83] show promising anticancer activity. However, they still require further evaluation before entering clinical trials. 

Natural substances, such as dietary flavonoids, polyphenols found mostly in fruit, vegetables, and other plant sources [84,85], have been largely studied for their beneficial role in inhibiting tumor development through the control of AhR activity [86,87,88]. Flavonoids induce apoptosis and cell-cycle arrest, the inhibition of metabolizing enzymes (notably cytochromes P450), the formation of reactive oxygen species (ROS), and the promotion of angiogenesis [89]. Several phase II clinical trials using flavonoids for cancer treatment have already been conducted for colorectal [90], breast [91], and prostate [92] cancer and melanoma [93]. However, their clinical use is limited due to inherent constrains, including their isolation/purification and pharmacokinetic challenges (e.g., bioavailability, drug–drug interactions, and metabolic instability) [89,94]. 

Urolithins (UroA), gut microbiota-derived metabolites of the natural polyphenol ellagic acid, have been shown to antagonize AhR [95] and induce senescence in human colon cancer cells [96] and prostate cancer [97]. Finally, various drugs used for purposes other than treating cancer display AhR-antagonist activity. These FDA-approved molecules could therefore be repurposed for cancer treatment. For example, clofazimine, an anti-leprosy drug, has shown clinical benefit for patients with multiple myeloma [98].

The disruption of AhR activity can be obtained by targeting the HSP90/p23/XAP2/AhR cytosolic complex. HSP90 inhibitors (XL888 or ganetespib) induce the degradation of their client proteins, including AhR. Escalating doses of HSP90 inhibitors in combination with a BRAF inhibitor (vemurafenib) was shown to increase the overall survival of BRAF V600E-mutated melanoma patients [99,100]. As HSP90 inhibitors show a very broad spectrum of action [101], degradation of AhR can be optimized by targeting the co-chaperone protein p23. Down-regulation of the p23 protein triggers ubiquitination of AhR [102] and specific inhibition of p23 (ailanthone) shows important anticancer effects in vitro [103]. 

Finally, the possible use of IDO inhibitors in cancer treatment has received much attention [39]. Although such treatment does not directly target AhR, they likely reduce kynurenine production and thus lower resistance to immune-checkpoint inhibitors [104]. 

To date, only two phase 1 clinical trials have been initiated to test direct modulation of AhR in cancer. The first, a non-randomized clinical trial conducted by Bayer^®^ (Leverkusen, Germany), aims to assess the tolerability and toxicity of an AhR inhibitor, BAY2416964, on 114 patients with advanced solid tumors and no therapeutic options (lung cancer, head and neck cancer, and colorectal cancer) (NCT04069026). Ikena Oncology^®^ (formerly Kyn Therapeutics^®^) (Boston, MA 02210, United States) also started a phase 1 non-randomized, open label, clinical trial in December 2019 to determine the tolerability and toxicity of KYN-175, an AhR inhibitor, on 53 patients with advanced solid tumors (NCT04200963). The first results of these two clinical trials are expected at the end of 2022. These trials underscore the importance of considering AhR as a next-generation cancer treatment. It is also worth considering targeting AhR as a complementary therapy, in combination with currently used treatments (i.e., targeted therapies and immunotherapies).

#### 2.3.4. AhR-Correlated Gene Signatures to Refine AhR-Targeted Therapy

**Figure 3:**
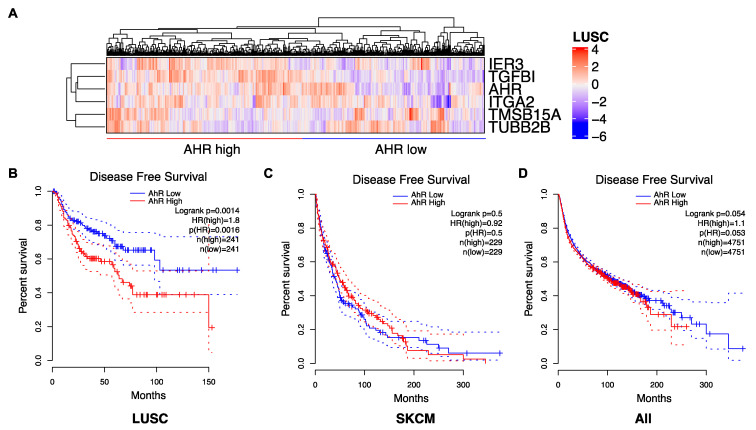
AhR correlated gene signature in LUSC. (**A**) Expression heatmap showing the expression of several AhR-correlated genes in patients with squamous-cell lung cancer (*n* = 486). Genes and clusters with similar expression profiles across the cohort are placed close to each other in the grid. (**B**–**D**) Disease-free survival curves for LUSC cancer patients (**B**), skin cutaneous melanoma (SKCM) patients (**C**), and all cancer patients from the TCGA (**D**), depending on the AhR signature corresponding to genes previously identified as the most positively and negatively (*n* = 48) correlated in lung-cancer cell lines.

Because the role of AhR in cancer is complex, we propose to tailor the AhR therapeutic strategy by considering the level of *AhR* expression (high/low) ((Figure 1B) and its correlated gene-signatures that are specific to tumor types (Figure 2A) and patients (Figure 3A). For example, an AhR high-signature is associated with an unfavorable prognosis, whereas an AhR low-associated signature is associated with a favorable prognosis in lung squamous-cell carcinoma (LUSC) (Figure 3A,B). As anticipated, a lung-specific AhR-correlated signature was not discriminative in terms of survival for patients with other tumors, such as skin cutaneous melanoma (SKCM) (Figure 3C), or transposable to all tumors (Figure 3D). Thus, one could choose to either antagonize or activate AhR according to an AhR-specific associated gene signature and patient outcome. 

#### 2.3.5. AhR as a Prognostic Marker to Choose the Most Efficient Targeted Therapy

Another possible strategy is to consider the level of *AhR* expression and that of its activity (expression of correlated genes) as a surrogate marker for new putative cancer therapies.

**Figure 4:**
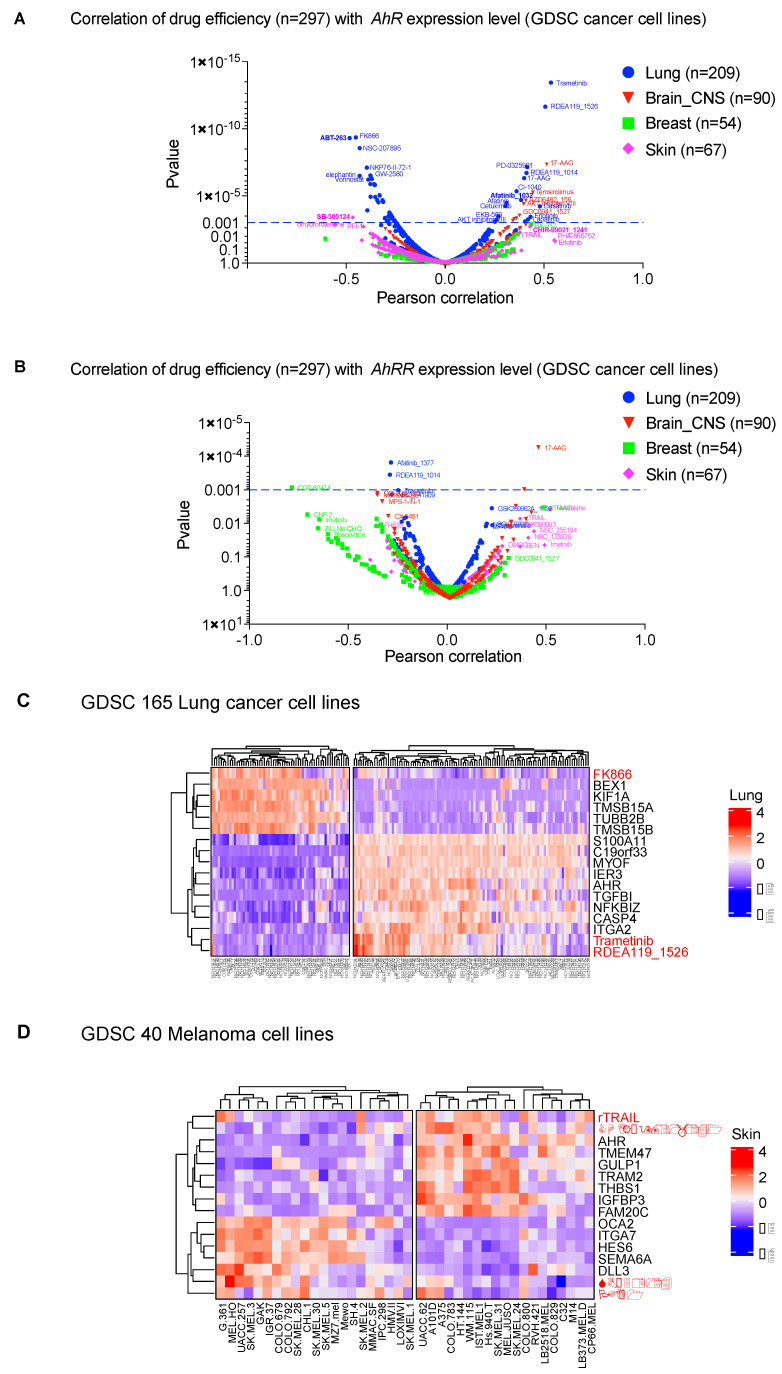
AhR signatures to identify therapeutic strategies in various cancer cell lines. (**A**) Volcano plot showing the correlation of drug efficiency (IC50) in various cancer cell lines (lung, brain-CNS, breast, skin) from the GDSC database (Genomics of Drug Sensitivity in Cancer, https://discover.nci.nih.gov/cellminercdb) with normalized level of *AhR* mRNA (RNAseq data). Drugs that are the most efficient when the *AhR* level is low are shown on the left, whereas drugs that are the most efficient when the *AhR* level is high are shown on the right. (**B**) Volcano plot showing the correlation of drug efficiency (IC50) in various cancer cell lines (lung, brain-CNS, breast, skin) from the GDSC database (Genomics of Drug Sensitivity in Cancer, https://discover.nci.nih.gov/cellminercdb) with the level of AhR regulator (*AhRR)* mRNA. (**C**,**D**) Expression Heatmap showing expression of the genes that correlate the most (positively or negatively) with that of AhR in lung-cancer cell lines (**C**) and skin melanoma cell lines (**D**) in terms of selected therapies for which the efficiency correlates with the level of *AhR* expression. Genes and clusters with similar expression profiles across the cohort are placed close to each other in the grid.

We explored this strategy by establishing the correlation of *AhR* mRNA levels and the therapeutic efficacy of 300 molecules (IC50) in various cancer cell lines (lung, brain-CNS, breast, skin) from the GDSC database (Genomics of Drug Sensitivity in Cancer, https://discover.nci.nih.gov/cellminercdb) (Figure 4A). We observed a significant correlation (*p* < 0.001) between drug efficiency (IC50) and *AhR* mRNA level. The correlation was specific for each tumor, with the highest correlation in lung-cancer cell lines (Figure 4A). Such a correlation analysis makes it possible to identify, among already available molecules, those that are adapted to the tumor type according to *AhR* level. For example, ABT-263 (Navitoclax), which targets the apoptosis inhibitor Bcl-2 [105], was more effective in lung-cancer cell lines that weakly express *AhR* (left panel of Figure 4A). The MEK inhibitor trametinib was more effective in lung-cancer cells that strongly express *AhR* (right panel of Figure 4A). Importantly, there was no correlation with expression of the AhR regulator (*AhRR*) (Figure 4B) [37].

**Figure 5:**
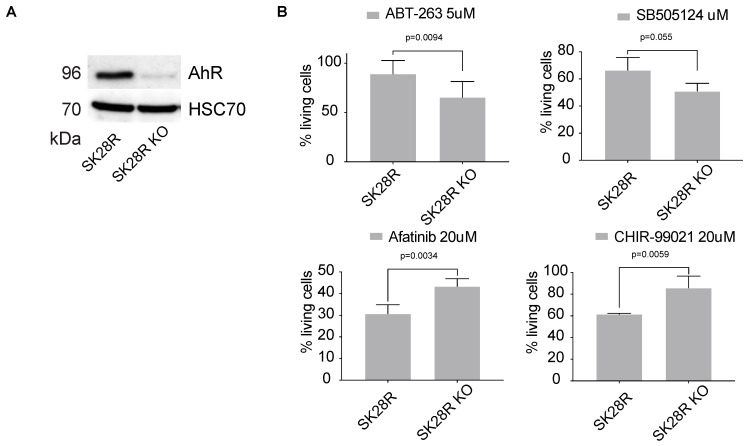
Drug efficiency in SKMel28 melanoma cell lines in the presence or absence of AhR. (**A**) AhR protein levels relative to that of HSC70 were analyzed by western blotting in SKMel28 cells in the presence or not AhR (CRISPR/Cas9). (**B**) SKMel28 and SKMel28 AhR KO cells were treated for 48 h at a dose leading to an approximately 50% reduction in cell viability (IC50). Histograms show the percentage of cell viability (*n* = 3) after treatment with ABT-263 (5 μM), SB505124 (20 μM), Afatinib (20 μM), and CHIR−99021_1241 (20 μM). Each histogram represents the mean ± s.d.; with unpaired *t*-tests with Sidak-Bonferroni method (*n* = 4–6).

In addition to the level of *AhR* expression, AhR-correlated signatures can also be considered to evaluate the potential effectiveness of a treatment. Indeed, we found that these gene signatures correlate with the efficacy of molecules previously highlighted in Figure 4A in lung-cancer cell lines (LUSC) (Figure 4C). Comparable results were obtained with melanoma cell lines (Figure 4D). We performed additional in vitro studies to validate the effectiveness of the inhibitors showing strong correlation with AhR level (Figure 4). Thus, we tested ABT-263, SB505124, Afatinib, and CHIR−99021_1241 on the melanoma line SKMel28 in the presence, or not, of the AhR transcription factor (CRISPR-Cas9 silencing) (Figure 5A). Briefly, SKMel28 and SKMel28 AhR KO cells were treated for 48 h at a dose leading to an approximately 50% reduction in cell viability (IC50). ABT-263 (5 μM) and SB505124 (20 μM) were more effective in the absence of AhR (SKMel28 AhR KO) (Figure 5B). Conversely, Afatinib (20 μM) and CHIR−99021 (20 μM) were more effective in the presence of AhR (SKMel28) (Figure 5B). These results are consistent with those obtained in Figure 4 showing the correlation between the sensitivity of different tumor cell lines to different treatments as a function of the level of expression of AhR and AhR-correlated genes. They thus reinforce the interest of analyzing both the level of *AhR* expression and the correlated transcriptional signature to define specific anti-tumor strategies.

#### 2.3.6. AhR as a Sensitizer of Cancer Therapies

The role of AhR as a sensitizer of existing targeted cancer therapies has thus far been little studied. In this context, in addition to FDA-approved targeted therapies, it is also possible to consider either promoting or inhibiting the AhR signaling pathway using agonists or antagonists, respectively. We have already reported such a strategy in the treatment of metastatic melanoma with BRAF V600E/K inhibitors (BRAFi). We showed that the acquisition of BRAFi resistance is accompanied by a strong induction of an AhR signature in cell lines and patients. An AhR antagonist, such as resveratrol, increased BRAFi sensitivity and delayed relapses in PDX melanoma [106]. Similarly, Yamashita et al. demonstrated that AhR counteracts the efficacy of doxorubicin (DOX) via enhanced AKR1C3 expression in TNBC through extensive metabolization of the drug. The cytotoxic effect of DOX was more pronounced in AhR^−/−^ MDA-MB 231 TNBC cells [107].

Genetic and metabolic alterations in basal-like and BRCA1-associated breast cancer can lead to chronic high levels of ROS, increasing the level of AhR protein and its transcriptional activity. Under these conditions, the AhR−AREG (Amphiregulin) signaling pathway positively supports tumorigenesis by controlling ROS and shaping the pro-tumorigenic functions of the tumor microenvironment. Given the effect of AhR inhibition on AREG levels and EGFR phosphorylation, synergistic effect of AhR inhibition together with EGFR inhibitor (Erlotinib) has been explored and showed a promising combinatorial antitumor effect [108].

In 2012, Barretina et al. created the “Cancer Cell Line Encyclopedia”, grouping the expression data of 947 human cancer cell lines, along with their respective sensitivity to 24 antineoplastic therapies [109]. They found that *AhR* expression was associated with the efficacy of MEK inhibitors in *NRAS*-mutant melanoma cell lines. Silencing of *AhR* suppressed the growth of *NRAS*-mutant melanoma cells expressing high levels of AhR. This finding underscores their growth dependency on AhR function. The study also highlighted the potential role of several MEK inhibitors as AhR antagonists. Overall, these results suggest that MAPKinase activation may co-occur with AhR-dependency and that elevated AhR levels may serve as a biomarker of sensitivity to MEK inhibitors in the context of *NRAS*-mutant melanoma.

The role of AhR in modulating the response to treatment has been more widely studied in the context of cancer immunotherapy and the IDO/TDO/Kyn pathway, linking AhR to the immune response [110,111]. IFN-γ induces tumor-repopulating cells (TRCs) to enter dormancy and escape immune surveillance through an IDO/TDO/Kyn-dependent pathway [112] Blocking IDO/AhR abrogates IFN-γ-induced dormancy and decreases tumor growth through inhibition of the STAT3/p53 pathway [113,114]. Treatment with tyrosine kinase inhibitors (TKis) (Dasatinib) can also counteract the effect of IDO to induce tolerogenic DCs in the tumor microenvironment. TKis could be used to modulate DC immunogenic activity and may potentially be applied to DC-based cancer immunotherapy as a complement to AhR or IDO inhibitors [115].

Although clinical trials targeting AhR for cancer are still very rare, the number of trials targeting the IDO/TDO/Kyn pathway has reached 100. These trials (4 in phase 1, 8 in phase 2, 9 in phase 3) are using IDO inhibitors (Epacadostat, Indoximolod, GDC-0919; etc.) in combination with immunotherapy (anti-PD-1: nivolumab or pembrolizumab, anti-CTLA4: ipilimumab, etc.) or targeted chemotherapies on different types of cancer (lung, breast, pancreas, etc.). It is reasonable to envisage complementary therapeutic trials directly targeting AhR and the IDO/TDO/Kyn pathway.

#### 2.3.7. AhR as a Drug Target to Counteract Resistance to Targeted Therapy

The development of resistance mechanisms to targeted therapies considerably limits the outcome for patients in the treatment of cancer. We recently implicated the AhR transcription factor in the acquisition of such resistance mechanisms following its increased activation. We showed that sustained activation of AhR induces the expression of genes associated with resistance to BRAF inhibitors in the treatment of metastatic melanoma [106].

**Figure 6:**
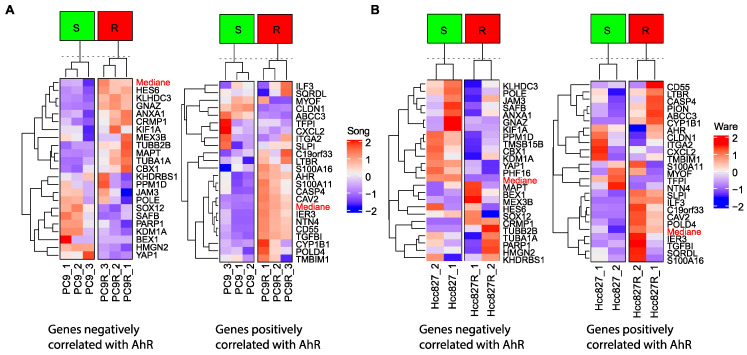
AhR signatures in tyrosine kinase inhibitor (TKi)-resistant (gefitinib) lung-cancer cell lines. (**A**,**B**) Expression heatmap showing the expression of genes that correlate the most highly (positively or negatively) with *AhR* mRNA levels (Figure 2A) in lung-cancer cell lines sensitive or resistant to a TKi (Gefitinib) from the data sets of Song et al. [117] (**A**) and Ware et al. [118] (**B**). Genes and clusters with similar expression profiles across the cohort are placed close to each other in the grid.

Similarly, AhR mediates the activation of PI3K/Akt and MEK/ERK signaling via Src kinase and induces resistance of EGFR-mutant NSCLC cells to an EGFR-TKi (Gefitinib) [116]. In this context, we analyzed the expression data of various lung-cancer cell lines that are sensitive or resistant (PC9 and Hcc827, respectively) to EGFR TKi (Figure 6A—data from Song et al.) [117], (Figure 6B—data from Ware et al.) [118]. We established expression signatures of genes that positively or negatively correlate with *AhR* expression (Figure 2). Such correlated AhR-signatures that classify sensitive and resistant cells could be used as markers of TKi resistance (Figure 6A,B). 

In addition, high doses of AhR ligand aminoflavone (AF) acts as an AhR antagonist, inhibiting Src-Akt signaling and suppressing α6-integrin expression to attenuate tamoxifen-resistance in MCF-7 breast cancer cells [119].

Histone deacetylase inhibitors (HDACis) (Aza-PBHA) are now widely used in anti-cancer treatment. However, they are largely ineffective against late-stage cancer due to acquired drug resistance and their relatively low specificity. Aza-PBHA increases PKCα phosphorylation and histone acetylation levels in human gastric-cancer cells by facilitating the interaction of HDAC with AhR. Thus, the use of PKCα inhibitors to control AhR-related epigenetic regulation is a promising potential method to prevent acquired resistance to HDACi-based cancer treatments [120].

It is also possible to control AhR protein levels in the context of resistance. He et al. have shown that ailanthone, which targets the co-chaperone protein p23, overcomes MDV3100 resistance in castration-resistant prostate cancer [121].

Overall, these studies show that it is important not only to analyze the level of AhR and its activity but also its correlated gene signature and pathway in the context of resistance to potentiate targeted therapies.

**Figure 7:**
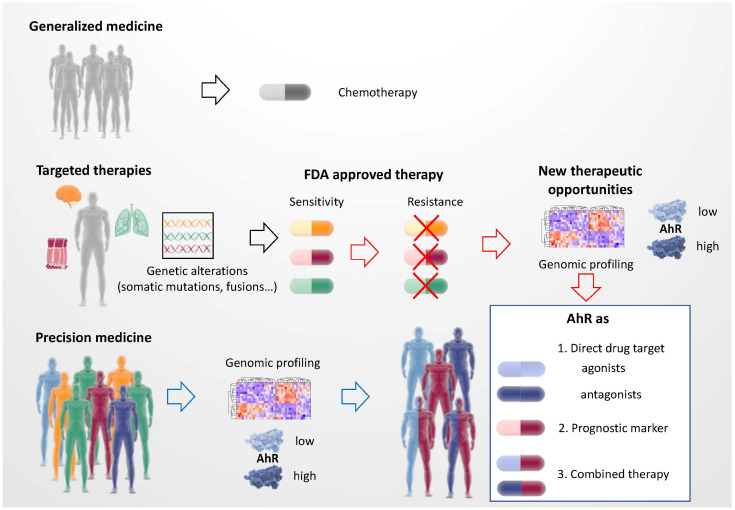
Precision medicine and the personalized therapy of cancer.

In conclusion, major advances in the identification of genetic alterations (somatic mutations, fusion transcripts, amplifications, deletions, etc.) have made it possible to shift cancer treatment from generalized chemotherapy (DNA alkylating agents, anti-mitotics, etc.) to targeted therapies (kinase inhibitors, immune checkpoint inhibitors) [122] (Figure 7). This has significantly improved patient survival through the use of monotherapy and combinatorial therapy.

Here, we have proposed several therapeutic strategies for the treatment of cancer in the context of precision medicine that can be applied by considering the level and activity of the AhR transcription factor (Figure 7). In the best situation, the targeted therapy is efficient in the long term and the patient shows complete tumor regression. However, most patients show short-term responses, followed by the appearance of resistance mechanisms, limiting the therapeutic benefit. Triggering AhR may constitute a promising option. AhR can first be considered as a direct drug target using AhR agonists or antagonists based on its level of expression and activity (AhR signature). In precision medicine settings, AhR could also be considered as a prognostic marker for identifying new putative therapeutic molecules to be used alone or in combination with AhR agonists or antagonists during the course of treatment. Finally, in the context of resistance mechanisms associated with AhR (deregulation of the AhR signature), it is possible to consider the use of new inhibitors (alone or in combination with AhR agonists/antagonists) to both sensitize therapy and prevent or slow the development of resistance. Overall, triggering of AhR for cancer treatment shows great potential.

## 3. Methods

### 3.1. Reagents

The inhibitors used in the study were as follows: Navitoclax (ABT-263) (Selleckchem, Houston, TX 77054 USA, S1001), Afatinib (BIBW2992) (Selleckchem, S1011), SB505124 (Selleckchem, S8523), and CHIR-99021 (Selleckchem, CT99021).

### 3.2. Cell Culture and Reagents

Human melanoma cell lines (SK28 and 501 Mel) were grown in humidified air (37 °C, 5% CO_2_) in RPMI-1640 medium (Gibco BRL, Invitrogen, Paisley, UK) supplemented with 10% fetal bovine serum (Eurobio, Les ULIS, France) and 1% penicillin-streptomycin antibiotics (Gibco, Invitrogen, Carlsbad, CA, USA). SK28 cells were obtained from the laboratory of J.C Marine at the VIB (Vlaams Instituut voor Biotechnologie) Center for Cancer Biology, VIB, Leuven, Belgium. All cell lines were routinely tested for mycoplasma contamination.

### 3.3. CRISPR/Cas9 Experiment

The AhR knockout was performed using CRISPR/Cas9 methodology. The guide sequence targeting AhR (Sigma-Genosys, St. Louis, MO, USA) was cloned into the GeneArt CRISPR Nuclease vector according to the manufacturer’s instructions (Life Technologies, Saint-Aubin, France). Next, vectors were transfected into SK28 cells and the cells seeded into 96-well plates two days later at 0.5 cells/well for single-cell clonal expansion. Clones of interest were validated by DNA-sequencing, western-blot analysis, and RT-qPCR [106].

### 3.4. Evaluation of Cell Density

Cell density was assessed using the methylene blue colorimetric assay. Briefly, cells were fixed for at least 30 min in 95% ethanol. Following ethanol removal, the fixed cells were dried and stained for 30 min with 1% methylene blue dye in borate buffer. After four washes with tap water, 100 μL of 0.1 N HCl was added to each well. Plates were next analyzed with a spectrophotometer at 620 nm.

### 3.5. Western Blot

Protein samples were denatured at 95 °C, resolved by SDS-PAGE, and transferred onto Hybond™-C Extra nitrocellulose membranes (Amersham Biosciences, Bucks, UK). Membranes were probed with the appropriate antibodies and the signals detected using a Fujifilm LAS-3000 Imager (Fuji Photo Film, Tokyo, Japan). The primary antibodies were anti-AhR (A3) and Hsc70 (B6) (Santa Cruz Biotechnology, Santa Cruz, CA, USA). Horseradish-Peroxidase-conjugated secondary antibodies were purchased from Jackson ImmunoResearch (Suffolk, UK) and used at a dilution of 1:10,000.

### 3.6. Data Mining

Meta-analysis from TCGA (The Cancer Genome Atlas) [123] and the GTEx (Genotype-Tissue Expression) [124] was performed and visualized using the publicly accessible web server GEPIA2 (http://gepia2.cancer-pku.cn). GEPIA2 is an updated version of GEPIA for analyzing the RNA sequencing expression data of 9736 tumors and 8587 normal samples from the TCGA and the GTEx projects, using a standard processing pipeline. GEPIA2 provides customizable functions, such as tumor/normal differential expression analysis, profiling according to cancer type or pathological stage, patient survival analysis, similar gene detection, correlation analysis, and dimensionality reduction analysis. This tool was developed by Zefang Tang, Tianxiang Chen, Chenwei Li, and Boxi Kang of Zhang Lab, Peking University [125]. Gene expression between normal tissue and cancer is visualized by a bar plot or by pathological stage plotted in Stage plot. Overall or Disease-Free Survival have been visualized in all cancer datasets, depending on the level of *AhR* expression, by calculating the hazards ratio based on the Cox PH Model.

The search for mutations (mutations, amplifications, deletions, etc.) for the transcription factor AhR was carried out using bioinformatics of the open source tool cBioPortal for cancer genomics (http://www.cbioportal.org) from the collection of databases available for various types of cancer (180 studies of patients and cell lines) (http://www.cbioportal.org/datasets). For specific information about the tools used to call mutations and the filters that may have been applied, refer to the published manuscript [126,127].

Analysis of the GDSC (Sanger/Massachusetts General Hospital Genomics of Drug Sensitivity in Cancer) [128] RNAseq dataset was performed and recovered from the CellMinerCDB webtool (https://discover.nci.nih.gov/cellminercdb) [38]. CellMinerCDB is an interactive web application that simplifies access and exploration of cancer cell line pharmacogenomic data across different sources. This webtool allows the comparison of molecular and/or drug response patterns across sets of cell lines to search for possible associations. Pearson’s correlations with reported *p*-values (not adjusted for multiple comparisons) between *AhR* expression (Figure 4A) and the expression of all other genes or *AhRR* expression (Figure 4B) expression with drug activity (297 compounds) were recovered for different cancer cell lines (lung *n* = 209, brain *n* = 90, breast *n* = 54, skin *n* = 67).

The raw data count matrix from the RNA seq data was obtained from the GEO database for the previous experiments on the lung-cancer cell lines (sensitive or resistant to an EGFR inhibitor: gefitinib) GSE79688 [https://www.ncbi.nlm.nih.gov/gds/?term=GSE79688] [118] and GSE129221 [https://www.ncbi.nlm.nih.gov/gds/?term=GSE129221] [117].

The expression heatmap of differentially-expressed genes between samples was obtained for a log2-fold change using the ComplexHeatmap 2.0.0 [129] package in R/Bioconductor. Cluster-specific gene rankings were obtained by contrasting samples with the rest of the samples. The volcano plots for the correlation with expression or drug sensitivity were established using GraphPad PRISM 8.0.

## Data Availability

Not applicable.

## Abbreviations

MDPI	Multidisciplinary Digital Publishing Institute
DOAJ	Directory of open access journals
AhR	Aryl hydrocarbon Receptor
Aldh1a1	Aldehyde dehydrogenase 1 family, member A1
ARNT	AhR Nuclear Translocator
BaP	Benzo (a) pyrene
bHLH/LZ	Basic Helix-Loop-Helix/leucine zipper
BRAF	serine/threonine-protein kinase B-Raf
ChIP	Chromatin ImmunoPrecipitation
CD34	Cluster of Differentiation 34
CRISPR-Cas9	Clustered Regularly Interspaced Short Palindromic Repeats CRISPR associated protein 9
CYP1A1	Cytochrome P450, family 1, subfamily A member 1
CYP1B1	Cytochrome P450, family 1, subfamily B member 1
EGFR	Epidermal Growth Factor Receptor
EMT	Epithelial-Mesenchymal Transition
FDA	Food and Drug Administration
FICZ	6-formylindolo[3,2-b]carbazole
GDSC	Genomics of Drug Sensitivity in Cancer
Hsp90	Heat shock protein 90
KO	Knockout
IDO	indoleamine-2,3-dioxygenase
IL6	Interleukine 6
ITE	(2-(1’H-indole-3’-carbonyl)-thiazole-4-carboxylic acid methyl ester)
JAG1-NOTCH1	Jagged1 notch receptor 1
MAPK	Mitogen Activated Protein Kinase
MEK	Mitogen-activated protein kinase kinase
p23	p23 HSP90 co-chaperone
PAS	Per-ARNT-Sim family
PDX	Patient Derived Xenografts
SHH	Sonic hedgehog
SMAD3	Mothers against decapentaplegic homolog 3
c-Src	Proto-oncogene tyrosine-protein kinase Src
STAT3	Signal transducer and activator of transcription 3
TCDD	2,3,7,8-tétrachlorodibenzo-p-dioxine
TCGTCGA	The Cancer Genome Atlas
TDO	tryptophan-2,3-dioxygenase
TGF-β	Transforming Growth Factor β
XAP2	hepatitis B virus X-associated protein
XRE	Xenobiotic Response Elements
